# Aristolochic Acids: Newly Identified Exposure Pathways of this Class of Environmental and Food-Borne Contaminants and its Potential Link to Chronic Kidney Diseases

**DOI:** 10.3390/toxics7010014

**Published:** 2019-03-19

**Authors:** Chi-Kong Chan, Yushuo Liu, Nikola M. Pavlović, Wan Chan

**Affiliations:** 1 Department of Chemistry, The Hong Kong University of Science and Technology, Clear Water Bay, Kowloon, Hong Kong; ckchanak@connect.ust.hk; 2 Environmental Science Programs, The Hong Kong University of Science and Technology, Clear Water Bay, Kowloon, Hong Kong; yliuee@connect.ust.hk; 3 Clinic of Nephrology, Clinical Centre Niš, Niš 18000, Serbia; 4 Medical Faculty, University of Niš, Niš 18000, Serbia; 5 Serbian Medical Society, Branch Niš, Niš 18000, Serbia; 6 Division of Environment & Sustainability, The Hong Kong University of Science and Technology, Clear Water Bay, Kowloon, Hong Kong

**Keywords:** aristolochic acids, food contamination, environmental pollution, root uptake, aristolochic acid nephropathy, Balkan endemic nephropathy, chronic kidney disease

## Abstract

Aristolochic acids (AAs) are nitrophenanthrene carboxylic acids naturally produced by *Aristolochia* plants. These plants were widely used to prepare herbal remedies until AAs were observed to be highly nephrotoxic and carcinogenic to humans. Although the use of AA-containing *Aristolochia* plants in herbal medicine is prohibited in countries worldwide, emerging evidence nevertheless has indicated that AAs are the causative agents of Balkan endemic nephropathy (BEN), an environmentally derived disease threatening numerous residents of rural farming villages along the Danube River in countries of the Balkan Peninsula. This perspective updates recent findings on the identification of AAs in food as a result of the root uptake of free AAs released from the decayed seeds of *Aristolochia clematitis* L., in combination with their presence and fate in the environment. The potential link between AAs and the high prevalence of chronic kidney diseases in China is also discussed.

## 1. Introduction

**Figure 1:**
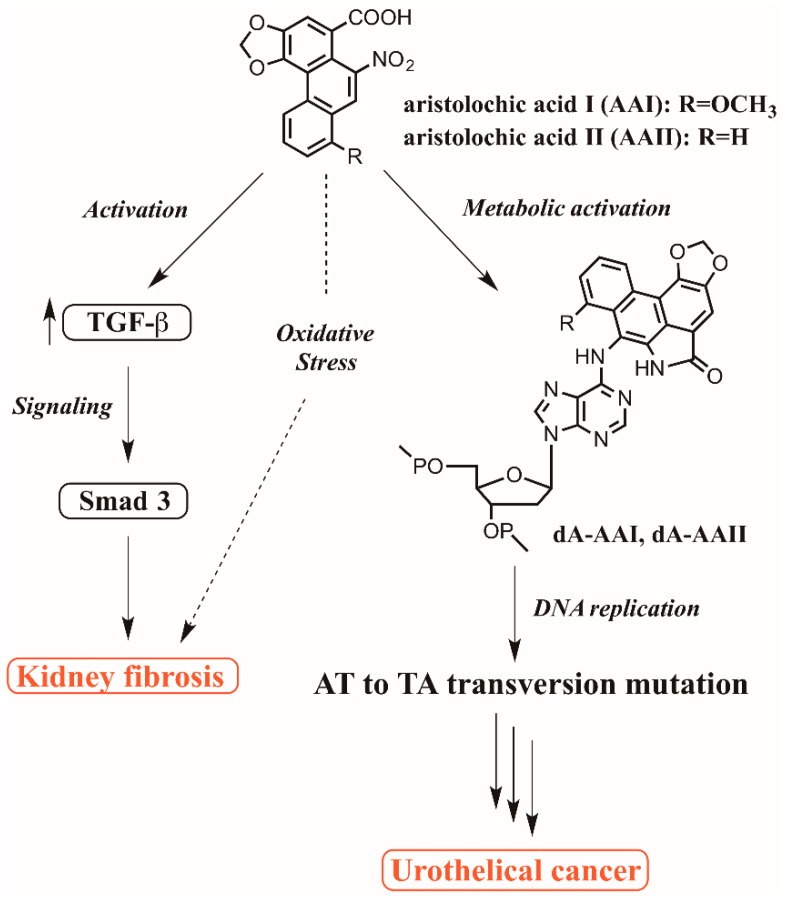
Postulated mechanisms for the nephrotoxicity and carcinogenicity of aristolochic acids.

Aristolochic acids (AAs, Figure 1) are a class of nitrophenanthrene carboxylic acids naturally produced in *Aristolochia* species (*spp.*) (notably *Aristolochia clematitis* L., *Aristolochia contorta* Bunge, *Aristolochia fangchi* Y. C. Wu ex L. D. Chow & S. M. Hwang, *Aristolochia debilis* Siebold & Zucc. and *Aristolochia manschuriensis* Kom.), *Bragantia spp.* or *Asarum spp.* plants that have been widely used as herbal medicines [1]. As early as 1964, Jackson et al. observed the nephrotoxicity of AAs when high doses were administered to humans [2]. Their human carcinogenicity was not confirmed until the diagnosis of tumors and, later, the identification of DNA-AA adducts (Figure 1) in animal models exposed to AAs and in AA-intoxicated patients [3,4,5]. The term “Chinese herbs nephropathy” (CHN) was used to describe this unique type of rapidly progressive nephropathy causing end-stage renal failure and urothelial cancer [6]. AAs and AA-containing herbs are currently classified by the International Agency for Research on Cancer as Group I carcinogens (carcinogenic to humans), and the sale of AA-containing herbal medicines is banned by countries worldwide [1,7].

The origin of CHN was the grim discovery of hundreds of Belgian women who developed severe renal diseases in 1992 after taking diet pills containing extracts of Chinese herbs [8]. Follow-up investigations revealed the presence of AAs in the diet pills and showed that *Stephania tetrandra* S. Moore (Han Fangji) had been inadvertently replaced by *A. fangchi* due to their common Chinese names (both Fangji) [9]. The claim was further supported by the detection of DNA-AA adducts in kidney tissues from the CHN patients that proved their previous exposure to AAs [10]. The term CHN has thus been revised to aristolochic acid nephropathy (AAN) for a more accurate description of the etiological agent of the disease. 

After the disclosure of cases in Belgium, similar cases linked to the intake of Chinese herbs were reported in different countries and regions, including Spain, Japan, France, the United Kingdom, Taiwan, the United States, Germany, China, Korea, Australia, Bangladesh, and Hong Kong [11]. In addition, Chinese herbs containing AAs were used as remedies for other purposes such as eczema and pain relief [12]. Although the use and retail sale of AA-containing herbs have been banned worldwide since the discovery of its toxicity, these cases, especially the recent ones [11], revealed that the general population can still obtain AA-containing remedies through online platforms and pharmacies without a prescription. 

**Figure 2:**
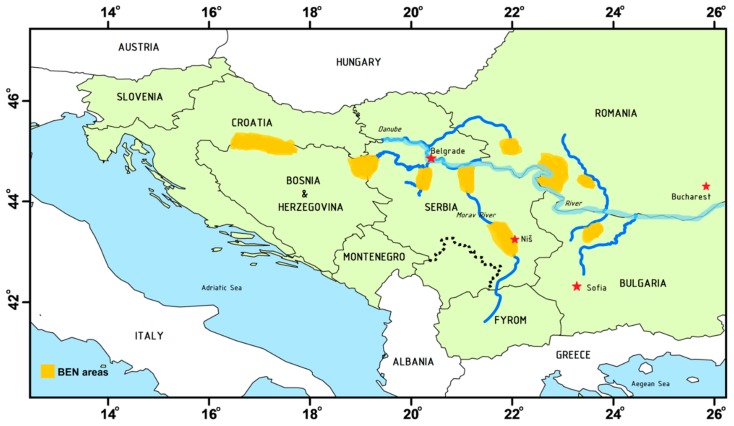
Map showing the geographic distribution of endemic nephropathy in rural farming villages located near tributaries of the Danube River in countries of the Balkan Peninsula.

For decades, residents in farming villages along the Danube River of the Balkan Peninsula have suffered from a mysterious end-stage renal disease, Balkan endemic nephropathy (BEN). A high prevalence of this chronic kidney disease (CKD) was observed among farmers in Bosnia and Herzegovina, Bulgaria, Croatia, Romania, and Serbia (Figure 2) [13,14]. The potential causative roles of polycyclic aromatic hydrocarbons (PAHs), heavy metals, and aflatoxins to the development of the disease were investigated. However, no conclusions could be drawn based on these studies. Since BEN shares common clinical features with AAN, it was later proposed that chronic dietary poisoning by AAs through contaminated food is responsible for the development of BEN. In 1969, Ivić speculated that the seeds of *A. clematitis* (commonly known as birthwort), a widespread AA-containing weed, intermingled with wheat grains during the machine harvesting process and contaminated the food [15]. However, the sources of human exposure pathways to AAs remained unknown for a long time.

Environmental exposure to AAs has been proposed to be the main cause of BEN. Only recently, through a collaborative effort led by research groups from Hong Kong and Serbia, scientists have further supported and strengthened the hypothesis that BEN is an environmentally derived disease, by analyzing wheat and corn grains cultivated in endemic villages in Serbia [16]. Both AA-I and AA-II were detected in the food grains and cultivation soil samples collected from the endemic villages, providing the first direct piece of evidence for this novel exposure pathway in the etiology of BEN [16]. It has been estimated that more than 25,000 individuals in the Balkan area are afflicted with BEN, and more than 100,000 individuals living in endemic regions could be at risk [17]. AA-associated renal diseases affect not only residents of the Balkan area but also residents of countries and regions where the practice of traditional Chinese medicine (TCM) is deeply rooted, such as China, Taiwan, and South Korea. It was projected that more than one hundred million Chinese are suffering from CKD, and some of the pathology and disease-causing agents have yet to be elucidated [17]. In this perspective, we claim that AA intake via AA-contaminated food is likely to be one of the primary causes of BEN. Moreover, we propose the novel concept that the consumption of AA-containing TCM could have been and still is the major pathway of human exposure to AAs and is responsible for CKD in China.

## 2. The Occurrence of Aristolochic Acids in the Environment

AA-containing species of *Aristolochia*, *Bragantia*, and *Asarum* usually grow in valley ditches, on roadsides and on hillsides as bushes at 200–1,500 meters above sea level. They can be widely found as weeds in tropical and subtropical areas, including continental Southeast Asia, Malaysia, China, tropical Africa, and South America. For instance, in China, AA-containing plants are known to grow in the south near the Yangtze River to the Yellow River Basin in Shandong and Henan provinces [18], and they are cultivated to prepare TCM in Guangdong and Guangxi provinces. Despite not yet being reported in the literature, it is possible that the nonmedicinal parts, e.g. leaf and root, of these plants may decompose in the soil and release free AAs into the environment.

**Figure 3:**
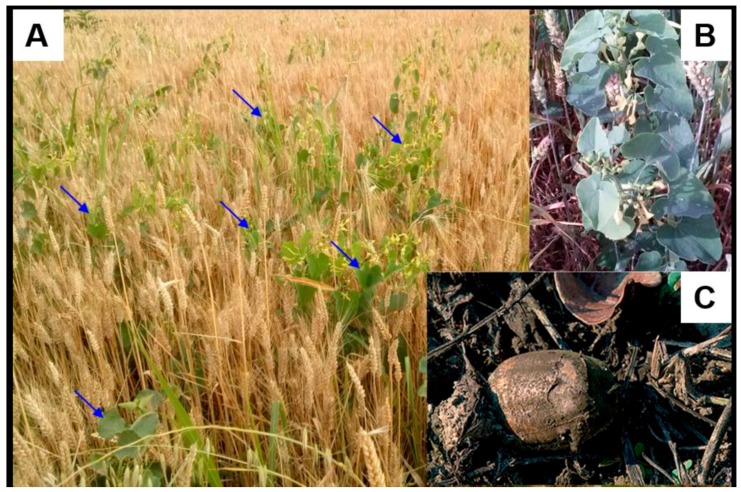
Photos showing (**A**) *Aristolochia clematitis* growing in a wheat field in the village Kutleš in Serbia (photo taken in May 2015), (**B**) enlarged view of *Aristolochia clematitis* growing together with wheat in cultivated field, and (**C**) decaying seed of *Aristolochia clematitis* in the wheat field.

Evidence of *Aristolochia spp.* in the Balkan Peninsula is also widely reported in the literature, and these plants are thought to be related to BEN [19]. Although environmental exposure to AAs has long been claimed to be the trigger for the mechanisms of BEN, specifically, in the hypothesis that the fruit of *A. clematitis* comingles with wheat grains during the harvesting process, no scientific evidence has been provided to support this claim [15]. Questions have been raised as to this proposal because of the different ripening times of wheat and *A. clematitis* in the Balkans (Figure 3); while wheat is ripe in early summer (end of June-beginning of July), the fruit of *A. clematitis,* which is rich in AAs, matures in late July [20,21]. Moreover, the significantly different sizes and weights between an *A. clematitis* fruit and wheat would result in most of the coharvested *A. clematitis* seeds being discarded during the threshing and milling processes [21]. Recently, free AAs released from the decay of *A. clematitis* were identified in the soil of a cultivated field in an endemic area of Serbia [16,22]. This identification raised the possibility that the agricultural soil in endemic areas throughout the Balkan region is extensively contaminated with AAs from the decay of *A. clematitis* plants, which are widespread in the endemic areas.

The possibility that water systems in the Balkan regions, including drinking and irrigation water, might be contaminated by AAs that originated from leaching soil is consistent with that finding. However, until now, there was no scientific evidence showing that irrigation water from a river or another water body could contain significant levels of AAs or other environmentally toxic agents released from soil runoff or other sources. The Pliocene lignite hypothesis, an attempt to explain the rise of BEN, postulates that chronic exposure to PAHs or other toxic organic compounds occurs because of leaching from the lignite low-rank coals underlying the endemic settlement into the deep well water [23]. It is not known whether AAs even exist in coal, originating either from the lignite structures and/or soil leaching, to leach into the water. Thus, the analysis of AAs in water bodies, such as wells and streams, is extremely important to public health.

An additional exposure pathway of AAs could result from environmental transport mediated by butterflies. Several species of *Papilionidae* protect themselves from predators by sequestering AA in their body and thus provide their own defense by becoming unpalatable. The larvae of *Papilionidae* in the *Zerynthiini* and *Troidini* tribes feed on *Aristolochia* containing AAs, which are then ingested and stored during the pupal and adult stage of the butterfly. *Zerynthia polyxena*, a butterfly that feeds on *A. clematitis*, accumulates AA-Ia and AA-C in its body tissues. *Battus polydamas cebriones*, belonging to the *Troidini*, which primarily feeds on *A. trilobata,* was found to release AAs in a sample of “Chiniy-trèf”, which is a type of medicine produced by maceration of the larvae of *Battus polydamas cebriones* [24].

## 3. Uptake and Bioaccumulation in Food Crops

**Figure 4:**
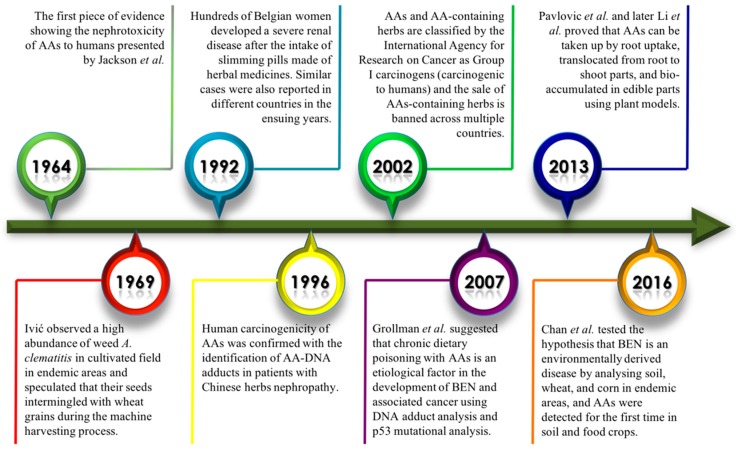
A brief timeline of the discovery of the toxicity of AAs and their occurrence in food and the environment.

Studies have shown that plants absorb and accumulate different environmental pollutants from the soil, such as heavy metals, antibiotics, and pesticides [25,26,27]. As Chan et al. have demonstrated, the existence of AAs in the cultivated fields in Serbia (Figure 4) [16] raises the concern of whether AAs translocated from the soil into food crops grown in contaminated fields could accumulate in the plants.

The fate and transport of AAs are difficult to predict, given their complex structures. The pKa values of AA-I and AA-II are 3.3 and 3.2, respectively [12], and as soil pH typically is between 5.5 to 8.0, the anionic forms of AAs dominate. The octanol-water partition coefficient (*K_ow_*) further describes the fate and transport of AAs. Previous studies recorded the log *K_ow_* values of AA-I and AA-II to be 1.65 and 1.23 at natural pH and found that the maximum translocation of chemicals from the soil medium occurs in chemicals with *K_ow_* ~1.78 [12]. These characteristics consequently create the possibility that AAs, AA-I in particular, could be taken up by the root, translocated from the root to parts of the shoot and bioaccumulated in the food grains. This hypothesis was further supported by the observation that significantly higher levels of AA-I than of AA-II were observed in wheat grain, tomato fruits, and spring onions.

This hypothesis was recently confirmed by Pavlovic et al. and Li et al., where the former used maize (*Zea mays*) and cucumber *(Cucumis sativus*) as plant models to demonstrate the highly efficient root uptake of AAs from a contaminated nutrient solution in a laboratory setting. The data confirmed that AAs can be absorbed from a nutrient solution by the roots of maize and cucumber [19]. The unequivocal presence of AAs in the roots provided the first line of evidence that root uptake may be one of the principal exposure pathways accounting for the etiology of BEN. To establish a direct relationship between the root uptake of AAs in food crops and the human exposure pathway to AAs in natural settings, Li et al. cultivated lettuce, tomatoes, and spring onions in contaminated soils to mimic realistic environmental exposure to AAs. The results provided the first line of evidence that AAs were transferred from contaminated media to edible parts of common food crops and that they were highly resistant to microbial activity and plant cell metabolism and were therefore able to persist in food crops [28].

Based on these findings, some researchers speculated that if the soil in some endemic areas contained residual AAs released from *A. clematitis*, it would also contaminate the food crops produced in the endemic villages. Therefore, Chan et al. collected soil, wheat, and corn grain samples from the well-known endemic village Kutleš in Serbia. By using high-performance liquid chromatography with fluorescence detection (HPLC-FLD), a method of high sensitivity and selectivity, AAs were detected in soil, corn and wheat samples for the first time [16]. The soil samples collected showed measurable concentrations of AA-I (86.59 ± 27.15 ng/g) and AA-II (25.73 ± 11.46 ng/g), and higher levels of AA-I (91.31 ± 89.02 ng/g) and AA-II (41.01 ± 12.45 ng/g) were detected in wheat grain. A similar observation was also recorded in Romania, where AA-I was detected in soil and soil organic matter samples retrieved from both endemic and non-endemic areas in the country [29], and in another large-scale study in Serbia [22]. These results further support the role of chronic environmental exposure to AAs in the etiology of BEN.

The prospect of finding AAs could be extended to other food crops, such as herbs and vegetables, that are grown in highly endemic regions and countries. It is not yet fully understood whether and to what extent the local and international food chains have been contaminated with AAs, although the prolonged consumption of food harvested from contaminated fields is presumed to be a leading cause of chronic intoxication, with AAs linked to the etiology of BEN and attendant upper tract urothelial cancers (UTUC). Thus, the extent to which nephrotoxic and carcinogenic AAs contaminate the environment remains a significant question to be addressed, suggesting the need to conduct worldwide surveillance of AAs in drinking water and food commodities.

## 4. Risks to Human Health

The European Agency for the Evaluation of Medicinal Products warns European Union member states “to take steps to ensure that the public is protected from exposure to AAs”. However, several publications reported that *A. clematitis* is a common weed in the cultivated fields in endemic areas, in addition to the original observation by Ivić [15,16,19,30]. These findings suggested that AAs could be released into the environment from the decomposition of the *A. clematitis* plant. In the village Kutleš (43°8’22.93″ N, 21°51’39.44″ E, elevation 206–214 m) in Serbia, widespread growth of *A. clematitis* in wheat and corn fields was observed, and the local soil and wheat samples showed a significant level of AAs (Figure 3) [16]. This finding further supported the idea that AAs can be taken up from the environment to contaminate the food crops and that AAs could enter human bodies and lead to BEN via the daily ingestion of AA-tainted food, thus causing public health problems.

A follow-up question could be whether the processing of wheat and maize flour can eliminate the AAs deposited in their respective grains. The typical method to produce flour is by milling, where grains are ground into flour. The other general procedures prior to milling include storing, cleaning, conditioning, and grinding. Cleaning merely removes coarse impurities and fine materials, which could include some fruits and parts of *A. clematitis*, but pollutants that have accumulated inside the grains are unlikely to be removed in the process. For instance, pesticides in wheat flour are often identified and quantified by academic scientists, and we cannot ignore the possibility that AAs contaminate local and international food chains through flour [31]. Similar to the research conducted to identify pesticides in flour samples, research to detect AAs in wheat and maize should be conducted to uncover and confirm the human exposure pathway to AAs and identify the links to BEN. Our group has recently developed methods to quantitate AAs in both wheat and maize flour, and we have collected locally produced samples from supermarkets in Serbia and Bulgaria. The preliminary results have shown that approximately one-fifth of the samples possess part-per-trillion levels of AAs (unpublished results). The positive identification of AAs in flour samples may directly threaten public health locally and globally. As the milling process cannot remove all of the AAs in grains, it is important to determine whether AAs could be eliminated by baking bread and cooking pasta. Methods to lower the quantity of AAs in flour and its products have been reported earlier, and show 30% of AA-I and 20% of AA-II can be removed from food after normal cooking methods such as baking and boiling. Notably, if cysteine is added to the food before boiling, 90% of AAs can be eliminated from AA-tainted food samples boiled in the water [32].

The carcinogenicity and nephrotoxicity of AAs have long been proven to threaten human lives, with numerous CHN, AAN and BEN sufferers identified globally. In addition to BEN, there are still some unknown chronic kidney diseases (CKD*u*), including Mesoamerican nephropathy (MeN), Sri Lankan CKD*u*, Indian CKD*u*, Egyptian CKD*u*, and Tunisian CKD*u*, occurring worldwide, and their etiological mechanisms have not been confirmed [33]. Due to the widespread distribution of *Aristolochia* and *Asarum* species worldwide and their widespread use as herbal medicines, it is possible that these CKD*u* could be linked to AAs at different extents. However, this claim has not been supported by any studies and is awaiting a more in-depth investigation.

## 5. Possible Metabolites in the Environment

The identification of AA metabolites in the environment, whether in soil or water systems, has not previously been reported. Interestingly, such metabolites have recently been found, for the first time, in the larva, pupa, and imago of Battus feeding on *Aristolochia* [34]. Some sequestered AAs in the Battus body (AA-Ia, AA-IIIa, AA-IVa, and AA-IVb) are converted to AA *O*-glucosides (AA-IaG, AA-IIIaG, AA-IVaG, and AA-IVbG) through insect-mediated *O*-glucosylation, in which the glucosyl group links to those acids, and is known to be the main detoxification pathway for the insects [34,35]. Notably, the larval integument contains a high concentration of AAs, while the adult contains a lower level than the larva, which indicates that molting is another method of detoxification. The integuments decompose and release AAs into the environment after metamorphosis [34]. Ultimately, the major sink of AAs occurs in the natural environment, such as water bodies and soil systems. 

Existing research [28] has raised the possibility that AAs may degrade in the soil, but their metabolites and the mechanism of their formation are still unknown, suggesting that the identification of AA metabolites merits additional research. It is hypothesized that experiments such as spiking AAs into a soil model could simulate the degradation of naturally existing AAs in the environment. After the soil incubation process, liquid chromatography coupled with mass spectrometry can be employed to analyze the extracts of the soil samples and identify AA metabolites.

## 6. Overview of Potential Biomarkers for AA intoxication

Cancer and nephropathy caused by AAs require the development of potential biomarkers to facilitate clinical diagnosis. The biomarkers known to date to assist in diagnosing AA nephropathy are classified into two categories, namely, biomarkers of exposure and biomarkers of effect.

Regarding biomarkers of exposure, carcinogenesis and mutagenicity were found to correlate with the formation of DNA-AA adducts [36]. Once AAs enter rodent or human bodies, they are first activated by enzymatic nitro-reduction to form *N*-hydroxyaristolactams and then become reactive cyclic *N*-acylnitrenium ions (Figure 1) [11]. The enzymes responsible for the nitro-reduction step are NAD(P)H:quinone oxidoreductase, hepatic microsomal cytochrome P450 (CYP) 1A1/2 and kidney microsomal NADPH:CYP reductase [37,38]. The delocalized positive charge on the *N*-acylnitrenium ion then attacks the exocyclic amino groups on dA, dG and dC to give stable and persistent DNA-AA adducts [37,38]. 7-(Deoxyadenosine-*N*^6^-yl)-aristolactam I (dA-AAI) is the most abundant DNA adduct formed and exhibits extended persistence in kidney (Figure 1) [39]. Other specific DNA-AA adducts, such as 7-(deoxyadenosine-*N*^6^-yl)-aristolactam II (dA-AA-II), 7-(deoxyguanosine-*N*^2^-yl)-aristolactam I (dG-AAI), and 7-(deoxyguanosine-*N*^2^-yl)-aristolactam II (dG-AA-II), were found in renal tissues of patients diagnosed with AAN and BEN [40,41]. A number of studies also identified the presence of these adducts in other organs, including the liver and stomach [40,42,43]. These adducts accumulate in cells and hinder DNA replication, eventually causing cell dysfunction and death. Chen et al. observed 151 cases of upper urinary tract epithelial cancer in Taiwan, where AA-laced herbal remedies are used extensively, and found that AAs reacted with DNA to form DNA-AA adducts, which have unique mutation characteristics in tumor suppressor gene *TP53* [44]. The oncogenes *FGFR3* and *HRAS* also seem to contain mutations induced by AAs, as the frequency of A:T crossover at codons 373 and 61 of *FGFR3* and *HRAS* in Taiwanese patients was five times higher than that in patients with urothelial cancer worldwide [45]. In addition, analysis of the data of UUC patients showed that the unique A:T to T:A transversion mutation formed mutation hotspots (53.1% of the total). *FGFR3* and *TP53* mutations define different approaches in the early diagnosis of urothelial cell carcinoma [46].

Another category of biomarkers is the biomarkers of effect. Li et al. discovered that oxidative stress participates in the development of AAN due to the significant decrease in the level of the antioxidant glutathione (GSH) [47]. For instance, they observed an elevated level of methylglyoxal (MGO) in kidneys in their mouse models, and the highly cytotoxic MGO could further modify proteins to form an advanced glycation end product, *N*^ε^-carboxymethyllysine (CML) [47]. The generation of MGO and CML in combination with reduced intrarenal antioxidant capacity can further lead to aging problems and diabetic complications [48]. In the treatment of AAN, we may consider controlling the levels of MGO and CML. A possible alternative mechanism of AA-induced toxicity is that AA-I depletes thiols in cells, whose imbalance impacts reactive oxygen species generation, DNA damage, and mitochondrial dysfunction [49]. AA-I is hydrogenated by reaction with cysteine or glutathione in cells, followed by loss of the nitro group. Because this reaction may occur under conditions similar to those in the human body (pH 7.0 and 37 °C), it is reasonable that AA-I may damage kidney cells by reacting away cysteine or GSH [50]. Cysteine is essential for many peptides and proteins, such as the antioxidant GSH and iron-sulfur cluster alloys [51,52]; GSH helps to maintain the normal function of the immune system and detoxifies some drugs (paracetamol), toxins (free radicals, iodoacetic acid) and heavy metals (lead and mercury) [53]. Therefore, AA-I may reduce thiol levels in the kidneys, leading to nephropathy caused by functional aggregation and oxidation.

## 7. Postulated Mechanism for AA-associated Kidney Fibrosis

One fundamental clinical feature of AAN is progressive kidney tubulointerstitial fibrosis, which is common in CKD and ultimately leads to end-stage renal disease [11]. AAs have been shown to cause lesions, leading mainly to renal tubular epithelial cell (RTEC) and tubulointerstitial injuries, in both in vitro and in vivo studies [54,55,56]. Under normal circumstances, these injured epithelial cells demonstrate a strong ability for repair through cell necrosis and apoptosis [54]; the surrounding intact cells then proliferate actively to maintain tubule integrity and renal function [54,57]. However, upon examination of the renal biopsy specimens from AAN patients, Yang et al. discovered that the acute epithelial cell injury caused by AA intoxication was not repaired by normal cell regeneration. Yang et al. then investigated expression of the epidermal growth factor (EGF), which plays an important role in cell repair, modulating cell proliferation and growth by binding to its receptor, in the specimens [54]. The results showed that EGF expression was suppressed in AAN patients and could account for the absence of RTEC regeneration.

Most of the AA-intoxicated rodent models pointed to a significant role of the overexpression of transforming growth factor-β (TGF-β), an essential cytokine in fibrogenesis and stimulator of myofibroblast activation, in the development of interstitial fibrosis, although the underlying molecular events remained poorly understood in past decades [54,58,59,60,61]. More recently, TGF-β was proven to induce fibrosis by Smad 3 stimulation under various conditions, such as bleomycin-induced pulmonary fibrosis, obstructive nephropathy, and liver and colon fibrosis [62]. Zhou et al. found AA-induced progressive renal failure and tubulointerstitial fibrosis in Smad3 wild type mice, but not in Smad3 knockout mice, indicating that Smad3 is a key factor in the development of AA-induced nephropathy (Figure 1) and that the specific deletion of TGF-β/Smad3 signaling may be a potential therapeutic target for chronic AAN [62]. Furthermore, Dai et al. reported that the loss of a negative regulator of TGF-β/Smad signaling, Smad 7, could account for the progressive renal injury observed in a mouse model of chronic AAN [63]. This proposal was validated by dosing a Smad7 knockout model with AAs, which resulted in enhanced renal injury progression. The restoration and ultrasound-mediated gene transfer of Smad7 in the knockout mice and injured kidney of wild-type mice, respectively, were later demonstrated to inhibit the further development of chronic renal nephropathy [63]. Tying together these findings with previous studies on other renal disease models highlighting the capability of Smad7 overexpression to attenuate kidney inflammation and fibrosis [64,65], Smad7 could play a protective role in the pathogenesis of chronic AAN.

Recent studies also have shown that BMP-7 inhibited epithelial to mesenchymal transition (EMT) through reducing the production of TGF-β and myofibroblast activation in HK-2 cells initiated by AAs [66]. EMT is associated with injury repair, tissue regeneration, and organ fibrosis. Its main biological function is to produce fibroblasts to repair tissue damage caused by trauma and inflammation. Additionally, the promotion of the AA-induced EMT in HK-2 cells was observed after the addition of gremlin, an antagonist of BMP-7 [67]. Even though several pathways of AA-induced TGF-β have been discovered, there is a gap in understanding the mechanisms of AA-induced renal fibrosis, such as the role of oxidative stress in kidney fibrosis (Figure 1). 

## 8. Challenges and Future Perspectives

The study of AAs in the pathophysiology of BEN and other related CKD*u* presents an array of challenges. There are official and sound medical records of BEN and UTUC in the Balkan regions but no data or records about AA-contaminated crops, soil, or water, presenting difficulties in analyzing and defining their geographical distribution. If there were proper records and statistics on the geographical distribution of BEN patients and AA contamination in addition to the medical records, we could target those areas with high BEN incidences and launch large-scale projects to discover the root cause of the diseases. This strategy could also be extended to countries that are suffering from CKD*u*.

AAN does not endanger only a single country or region, since cases have been reported in many countries worldwide in the past decades [11]. Due to the financial instability of developing countries and lack of evidence regarding the presence and geographical distribution of AAs in developed countries, the situation of AA contamination may not be easily improved. The identification of AAs in the environment and food samples can be challenging since the concentrations of AAs present in the samples are usually at trace levels within complicated sample matrices, and thus require highly sensitive instruments and skilled technicians. Therefore, acquiring instruments and employing experts to perform a comprehensive investigation of all potential sources of AAs in the environment could be costly for local governments, who may be reluctant to prioritize and confront an existing AA contamination problem. In addition, agricultural activities are often the backbone of the economic system in developing countries, and it may be infeasible to terminate the cultivation of food crops in the contaminated areas and implement soil remediation efforts. Additionally, there is no available standard remediation method for AA-contaminated soil clean-up, in contrast to the soil remediation methods for other types of pollution, as recommended by the USA Environmental Protection Agency. 

With significant evidence demonstrating that AAs are strong human carcinogens and potent nephrotoxins [1], considered now to be a worldwide problem threatening more than one billion people, regulatory agencies should be alerted to the potential existence of AAs and should classify them as new contaminants in both soil and food crops. This recognition is especially important for the governments of Asian countries with a long history of practicing Chinese herbal medicine, such as China. More than 100 million people in mainland China are estimated to be suffering from CKD [17], but the pathology and disease-causing agents are not fully understood.

Owing to the widespread use of *Aristolochia spp.* in the preparation of traditional herbal remedies, Grollman has speculated on an unrecognized linkage between AAs and global diseases [17]. He and his team conducted a molecular epidemiologic approach in Taiwan and revealed that nearly one-third of the population had been administered AA-containing remedies. A dose-dependent relationship between the consumption of AA-containing herbal remedies and the risk of developing renal cancer was also observed [17]. Such an epidemiologic study should also be carried out in other countries with frequent use of AA-containing herbs, such as Korea and China. In Korea, the prescription of AA-containing herbal medicine to patients by clinics was known to be a cause of local AAN cases, even after the prohibition of AA-containing ingredients in herbal medicine by the Korea Food and Drug Administration and the number of AAN cases was reported to be underestimated [68]. Given that some *Aristolochia spp.* are still allowed to be used according to the 2015 Chinese Pharmacopoeia [17] and the list of already marketed TCMs with AA-containing herbs was recently disclosed by the China Food and Drug Administration, part of the Chinese population could have been exposed to AAs through TCM intake. 

Could AAs, therefore, be responsible for the prevalence of CKD in China or of other CKD*u*? To answer this question, at the first stage, large-scale TCM and herbal medicine analyses are needed to differentiate AA-containing and non-AA-containing TCM and quantitate the concentration of AAs in these remedies. Furthermore, to uncover the true cause of the diseases, the clinical features of CKD*u* could be compared to those of AAN and BEN, and the analysis of a robust and sensitive biomarker, DNA-AA adducts, should be performed in patients with CKD, given their high persistence in target organs and unique mutation patterns. Other biomarkers, such as covalent blood protein adducts, should also be developed to obtain simple and routine clinical tests and screening for human exposure to AAs.

Since *Aristolochia* plants grow all over the earth in different geographic environments, worldwide surveillance for the existence of AAs in cultivation fields and in food and drug products is urgently needed. To safeguard public health, methods for remediating AA-contaminated farmland should be developed and implemented in a timely and appropriate manner in contaminated areas. The residents in the endemic areas should be immediately informed of the existence of AAs in their cultivated fields, food, and drug ingredients in order to stop the dietary intake of AAs through AA-contaminated food and to help prevent them from acquiring end-stage kidney disease and carcinoma of the upper urinary tract. Last but not least, we should investigate the molecular events involved in the carcinogenesis and fibrogenesis of AAN and CKD more vigorously to discover new therapeutic agents for appropriate disease prevention and treatment.
